# The Prediction of Pre‐Eclampsia Using Low Fetal Fraction in a Machine Learning Model

**DOI:** 10.1002/pd.70033

**Published:** 2025-11-27

**Authors:** Jinyuan Wang, Yuxiao Bai, Shengshan Huang, Yao Lin, Songdao Ye, Wangxiao Zhou, Shanshan Li, Ni Li, Minghua Jiang, Xiaoou Wang

**Affiliations:** ^1^ Department of Clinical Laboratory The Second Affiliated Hospital and Yuying Children's Hospital of Wenzhou Medical University Wenzhou China

**Keywords:** cell‐free fetal DNA, non‐invasive prenatal testing, pre‐eclampsia, XGBoost

## Abstract

**Objective:**

To investigate the association between low fetal fraction (FF) in non‐invasive prenatal testing (NIPT) and pregnancy complications or adverse pregnancy outcomes.

**Methods:**

Sixty‐four pregnant women undergoing NIPT at the Second Affiliated Hospital of Wenzhou Medical University between 13 June 2019 and 6 January 2023 had an initial NIPT failure due to low FF. Three cases were lost to follow‐up, leaving 61 cases in the failure group (Group A). Group A was subdivided into 37 cases with a valid result after redraw (Group A1) and 24 cases remaining unsuccessful after redraw (Group A2). Concurrently, 119 pregnancies with successful NIPT (normal FF, no fetal chromosomal abnormalities) were randomly selected as controls (Group C). Logistic regression and XGBoost models were established, and their area under the curve (AUC), sensitivity, and specificity were calculated and compared.

**Results:**

The incidence of pre‐eclampsia was significantly higher in Group A than in Group C (*p* < 0.05). No significant difference in the incidence of pre‐eclampsia was found between Groups A1 and A2. A logistic regression model incorporating FF predicted pre‐eclampsia with an AUC of 0.750 (95% CI: 0.639–0.860), sensitivity of 0.875, and specificity of 0.727. An XGBoost model incorporating 10 factors (FF, age, weight, BMI, gestational age, systolic/diastolic blood pressure at sampling, ART history, delivery history, heparin use history) demonstrated superior performance (AUC = 0.956, 95% CI: 0.868–1.000; accuracy = 0.944). The top three important factors were systolic blood pressure, diastolic blood pressure, and FF.

**Conclusions:**

Low FF in NIPT may indicate an increased risk of pre‐eclampsia. Regardless of the success of redraw, pregnancies with initial NIPT failure due to low FF warrant vigilance for pre‐eclampsia development. The XGBoost machine learning model demonstrates good efficacy for predicting pre‐eclampsia and has potential as an adjunctive prenatal screening tool for early diagnosis.

## Summary

- What's already known about this topic?
  ◦ Low FF may be associated with an increased risk of pregnancy complications and adverse outcomes including pre‐eclampsia.
- What does this study add?
  ◦ This study indicates that the XGBoost machine learning model predicted pre‐eclampsia with high accuracy, demonstrating its potential as a beneficial adjunct to prenatal screening for early diagnosis.

## 1 Introduction

The proportion of cell‐free fetal DNA (cffDNA) in maternal plasma is termed the fetal fraction (FF) [1]. FF is a critical parameter in non‐invasive prenatal testing (NIPT), determining overall test performance. It correlates positively with the detection rate and sensitivity [2] and ensures accurate clinical interpretation of results. Typically, an FF below 2%–4% (platform/method‐dependent [3]) limits test validity and constitutes NIPT failure. Recent studies suggest that low FF may be associated with an increased risk of pregnancy complications and adverse outcomes, including pre‐eclampsia, gestational hypertension, and fetal growth restriction. However, the predictive capacity of low FF for specific complication types remains inconclusive due to inconsistencies across studies [4, 5, 6, 7, 8]. This study analyzed cases of NIPT failure due to low FF at our center to investigate the predictive value of FF for pregnancy complications and adverse outcomes. We also established a prediction model to aid in predicting and preventing these complications in pregnancies with low FF and NIPT failure.

## 2 Materials and Methods

### 2.1 Patients and Design

A total of 17,632 pregnant women who underwent NIPT after providing informed consent at the Second Affiliated Hospital and Yuying Children's Hospital of Wenzhou Medical University between 3 June 2019 and 6 January 2023 were recruited. Maternal data (age, height, weight, gestational age (GA), conception mode (spontaneous/assisted reproductive technology (ART)), and gestation type (singleton/twin) were collected, and pregnancy outcomes were followed. Initial NIPT failed in 121 cases, including 64 due to low FF. After excluding 3 cases lost to follow‐up, 61 cases comprised the failure group (Group A). Group A was subdivided into 37 cases obtaining a valid result after redraw (Group A1) and 24 cases remaining unsuccessful after redraw (Group A2). Concurrently, a control group (Group C) of 119 pregnancies with successful initial NIPT (normal FF, no fetal chromosomal abnormalities) was randomly selected. The selection was performed using a computer‐generated random number sequence applied to the entire pool of eligible pregnancies who underwent successful NIPT during the study period.

### 2.2 Determination of Low FF

The sequencing data in our laboratory were processed using the NIFTY system. When FF falls below 3.5%, the unique reads required for analysis increase exponentially, limiting test validity [9]. Thus, FF < 3.5% was defined as low FF.

### 2.3 Statistical Analyses

Statistical analyses were performed using SPSS version 25.0. Normality was assessed for all variables. Continuous variables are presented as mean ± standard deviation (x̄ ± s) for normally distributed data or median (quartiles) [M(P25,P75)] for non‐normally distributed data. Student's t‐test or the Mann‐Whitney *U*‐test was used for comparisons between two groups for normally or non‐normally distributed data, respectively. The Kruskal‐Wallis test was used for comparisons between multiple groups. The chi‐square or Fisher's exact test was used for comparing proportions. Statistical significance was set at *p* < 0.05.

### 2.4 Modeling

A multivariable logistic regression model was constructed to adjust for potential confounders, including maternal age, BMI, and GA. Additionally, an XGBoost model (Python package) was implemented to investigate complex, non‐linear associations. The dataset was randomly divided into a training set (80%) and a hold‐out test set (20%). Hyperparameter optimization was conducted on the training set using GridSearchCV from the scikit‐learn library, incorporating an internal 10‐fold cross‐validation to robustly assess model generalizability and mitigate overfitting. The key hyperparameters identified as optimal were: learning_rate (0.2), max_depth (3), min_child_weight (1), and *n*_estimators (100). A full description of the parameter grid searched is provided in Supporting Information S1: Table S1. The final model performance was evaluated on the independent hold‐out test set. Receiver operating characteristic (ROC) curves were plotted, and the area under the curve (AUC), sensitivity, and specificity were calculated. Finally, the ranking of feature importance was derived from the final trained model's predictions on the training dataset only, ensuring an unbiased interpretation of influential features.

## 3 Results

### 3.1 Patient Characteristics

Group A and Group C differed significantly in weight and BMI (*p* < 0.05). No significant differences were found between Groups A1 and A2. Both Groups A1 and A2 had significantly higher weight and BMI than Group C (*p* < 0.05). No significant differences were observed between the three groups regarding age, GA, multiparity history, heparin medication history, or autoimmune disease history (Table 1).

**TABLE 1:** Comparison of clinical characteristics, pregnancy complications, and adverse pregnancy outcomes.

		Group C (*n* = 119)	Group A (*n* = 61)	*p*‐value (C vs. A)	Group A (*n* = 61)	
					Group A1 (*n* = 37)	Group A2 (*n* = 24)
Characteristics	Age	30.08 ± 5.13	31.57 ± 4.18	0.052	31.00 ± 4.01	32.46 ± 4.36
	Weight	58.00 ± 9.99	64.42 ± 12.57	**0.001**	62.89 ± 12.09^a^	66.77 ± 13.19^a^
	BMI	22.49 ± 3.60	25.41 ± 4.57	**< 0.001**	24.63 ± 3.99^a^	26.62 ± 5.20^a^
	Gestational age	14.71 (14.28∼16.14)	15.00 (14.43∼16.86)	0.294	15.00 (14.43∼16.28)	15.43 (14.86∼17.12)
	History of multiparity	55 (46.22%)	21 (34.43%)	0.129	13 (35.14%)	8 (33.33%)
	History of heparin medication	9 (7.56%)	4 (6.57%)	1.000	2 (5.41%)	2 (8.33%)
	History of autoimmune diseases	1 (0.84%)	2 (3.28%)	0.552	1 (2.70%)	1 (4.17%)
Pregnancy complications						
Pre‐eclampsia		1 (0.84%)	7 (11.48%)	**0.004**	4 (10.81%)^a^	3 (12.5%)^a^
Gestational hypertension		7 (5.88%)	5 (8.20%)	0.784	2 (5.41%)	3 (12.5%)
Gestational diabetes		22 (18.49%)	14 (22.95%)	0.479	7 (18.92%)	7 (29.17%)
Adverse pregnancy outcomes						
Prematurity		4 (3.36%)	4 (6.56%)	0.547	3 (8.11%)	1 (4.17%)
Abortion		1 (0.84%)	1 (1.64%)	1.000	1 (2.70%)	0
Fetal growth restriction		0	1 (1.64%)	0.339	1 (2.70%)	0
Fetal macrosomia		11 (9.24%)	1 (1.64%)	0.105	1 (2.70%)	0
Chromosomal abnormality		0	2 (3.28%)	0.114	0	2 (8.33%)^a^ ^,^ ^b^

*Note:* Data are presented as mean ± standard deviation, median (interquartile range), or *n* (%). Bold values indicate statistical significance (*p* < 0.05).

^a^ Statistically different compared with Group C (*p* < 0.05).

^b^ Statistically different compared with Group A1 (*p* < 0.05).

### 3.2 Pregnancy Complications and Adverse Pregnancy Outcomes

The incidence of pre‐eclampsia was significantly higher in Group A than in Group C (*p* < 0.05). No significant difference was noted between Groups A1 and A2. Both Groups A1 and A2 had significantly higher pre‐eclampsia incidence than Group C (*p* < 0.05). Comparing adverse pregnancy outcomes, only the incidence of fetal chromosomal abnormalities differed significantly, being higher in Group A2 than in the other groups (*p* < 0.05) (Table 1). The chromosomal abnormalities in Group A2 are detailed in Table 2.

**TABLE 2:** Fetal chromosomal abnormality results.

Pregnancy outcome	No.	Chromosome results
Fetal chromosomal abnormality	1	Chromosomal karyotyping results: “46,X,del(Y) (q11.21q11.23)”, Chromosomal microarray analysis (CMA): “arr[GRCh38]Yp11.2q11.2(2782383_10624004)x2, Yq11.21q11.23(11680249_28799654)x0”
	2	Chromosomal karyotyping results: “46,XX”, Chromosomal microarray analysis (CMA): “arr[GRCh37]7q11.21(62572576_64158560)x1,Xp22.33(2693466_3562464)x1,Yp11.31p11.2(2650424_6150392)x1,Yp11.2(7283030_9526559)x1”

### 3.3 Logistic Regression Analysis for Predictive Value

**FIGURE 1:**
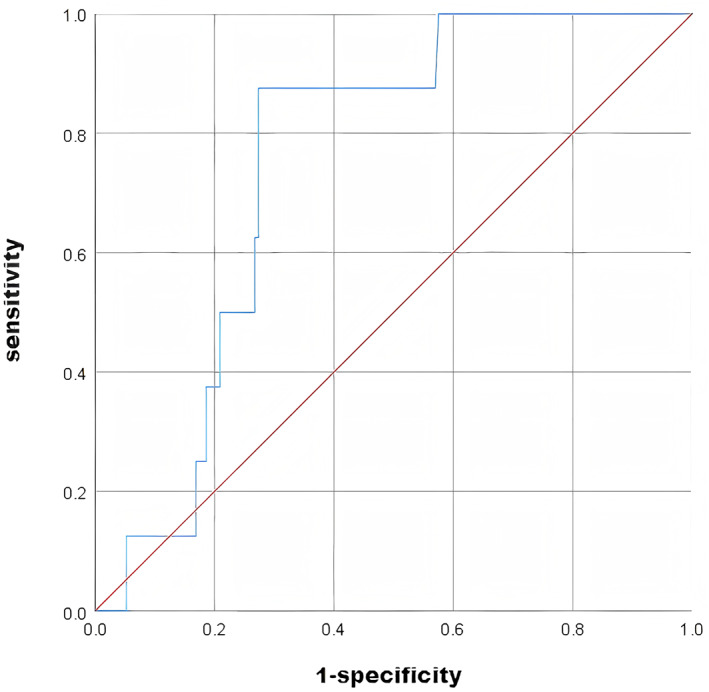
ROC curve of the univariate logistic regression model predicting pre‐eclampsia using FF. The logistic regression equation was *Y* = −1.328–0.302*FF. The model achieved an area under the curve (AUC) of 0.750 (95% confidence interval: 0.639–0.860), with a sensitivity of 87.5% and a specificity of 72.7% at the optimal cut‐off value.

Logistic regression including FF yielded the equation: *Y* = −1.328–0.302*FF. The model achieved an AUC of 0.750 (95% CI: 0.639–0.860), sensitivity of 0.875, and specificity of 0.727 (Figure 1). Using the optimal FF cut‐off of 3.396%, the pre‐eclampsia incidence was 11.29% (7/62) for FF < 3.396% and 0.85% (1/118) for FF ≥ 3.396% (*p* = 0.004).

To determine whether the association between low FF and pre‐eclampsia was independent of potential confounders, a multivariable logistic regression model was constructed. The model included FF, age, BMI, and GA as covariates. After adjustment, FF remained a significant independent predictor of pre‐eclampsia (adjusted odds ratio = 0.727, 95% CI: 0.553–0.958, *p* = 0.023). Notably, age, BMI, and GA were not significantly associated with the outcome in this adjusted model (*p* > 0.05) (Table 3).

**TABLE 3:** Multivariable logistic regression analysis of factors associated with pre‐eclampsia.

Variable	Adjusted OR (95% CI)	*p*‐value
Fetal fraction (%)	0.727 (0.553–0.958)	0.023
Age	1.019 (0.850–1.221)	0.837
BMI	1.010 (0.861–1.185)	0.905
Gestational age	0.574 (0.294–1.122)	0.105

### 3.4 XGBoost Model for Predictive Value

**FIGURE 2:**
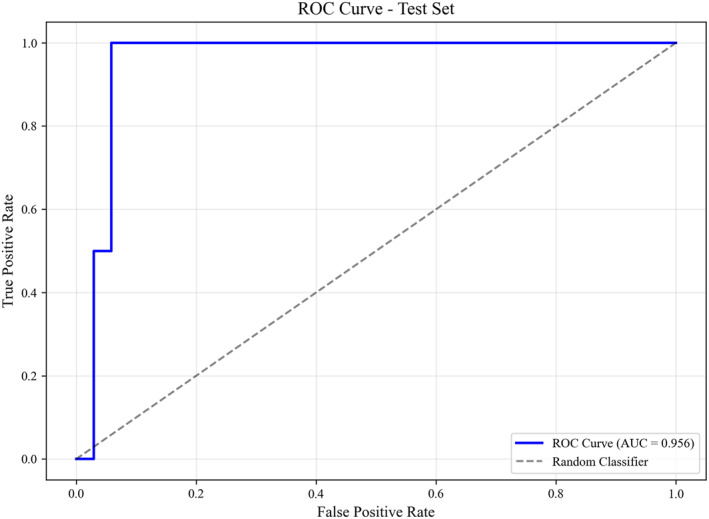
ROC curve of the multivariable XGBoost model for predicting pre‐eclampsia. The model incorporated 10 features: FF, age, weight, BMI, gestational age, systolic/diastolic blood pressure at sampling, ART history, delivery history, and heparin use history. The model demonstrates high predictive performance, with an AUC of 0.956 (95% CI: 0.868–1.000).

**FIGURE 3:**
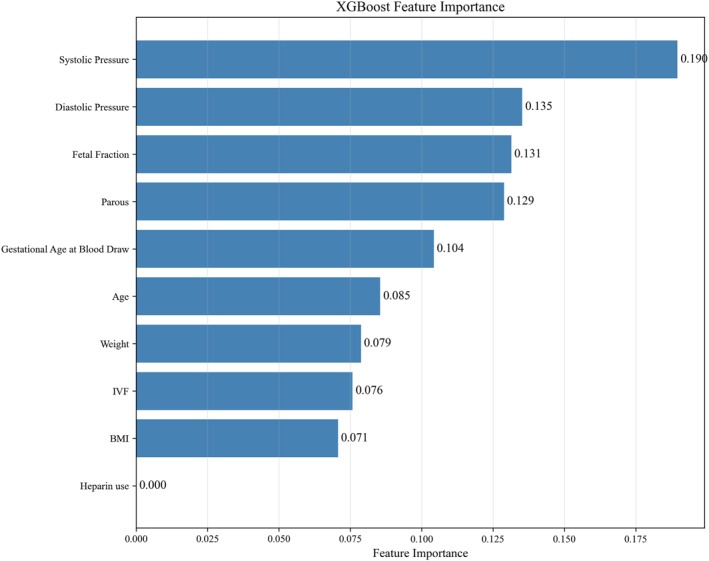
Feature importance ranking of the XGBoost model for predicting pre‐eclampsia. The plot displays the relative importance of each predictive variable in the final model, as determined by the XGBoost algorithm. The top three most influential features were systolic blood pressure at sampling, diastolic blood pressure at sampling, and FF.

An XGBoost model was developed using 10 clinical features—FF, age, weight, BMI, GA, systolic and diastolic blood pressure at sampling, ART history, delivery history, and heparin use history—to predict the risk of pre‐eclampsia. The model demonstrated excellent predictive performance, achieving an AUC of 0.956 (95% CI: 0.868–1.000) and an accuracy of 0.944 (95% CI: 0.861–1.000) (Figure 2). Bootstrap resampling with 1000 iterations further confirmed the model's robustness (Supporting Information S1: Table S2). Feature importance analysis revealed that systolic blood pressure at sampling, diastolic blood pressure at sampling, and FF were the three most influential predictors (Figure 3).

### 3.5 Follow‐Up of Pregnant Women With Pre‐Eclampsia

A total of eight pregnancies complicated by pre‐eclampsia were prospectively followed. Seven had low FF at the first blood draw. Among these seven, three demonstrated normalized blood pressure following redraw (two with normal FF levels upon redraw and one with persistently low FF); four had persistent hypertension (two with normal FF and two with persistently low FF upon redraw). One case presented with normal FF at the initial draw; however, the woman experienced elevated blood pressure during a hospital visit despite reporting normal readings at home (Table 4).

**TABLE 4:** Follow‐up results in pregnant women with pre‐eclampsia.

No.	FF at first blood draw (%)	FF at blood redraw (%)	Hypertension	Proteinuria	Pregnancy outcomes
1	3.395	6.84	144/99 mmHg	0.88 g/24h	Prematurity
2	3.358	3.294	126/99 mmHg	0.55 g/24h	/
3	3.004	4.18	147/88 mmHg	1.75 g/24h	/
4	3.373	5.77	153/104 mmHg	1.75 g/24h	Prematurity
5	2.043	2.591	149/95 mmHg	0.38 g/L	/
6	2.9	3.701	144/93 mmHg	0.46 g/24h	Abortion
7	3.197	2.18	144/99 mmHg	0.88 g/24h	/
8	9.63	/	160/109 mmHg	/	/

## 4 Discussion

The placenta is the primary source of cffDNA [10, 11, 12], suggesting that FF reflects placental status and potential abnormalities. Therefore, FF may serve as a marker for placental dysplasia/dysfunction associated with placenta‐derived complications. Early in pregnancy, extravillous trophoblast cells invade the uterus, transforming spiral arteries into low‐resistance vessels [7, 8]. Placental complications like pre‐eclampsia involve failed trophoblast infiltration and abnormal arterial transformation. This disrupts the placental‐maternal interface, reduces uteroplacental blood flow, decreases cffDNA release, and lowers FF [13], potentially explaining the link between low FF and adverse outcomes.

This study demonstrated a significantly higher incidence of pre‐eclampsia in both Groups A1 and A2 compared with Group C, supporting an association between low FF and complications. The lack of difference between A1 and A2 suggests that an increase in FF upon redraw exceeding the cutoff [14], while occurring alongside advancing gestation, may still represent a relative deficiency for their specific GA compared to normal pregnancies. In other words, although the absolute FF value in these pregnancies may have increased above the technical threshold due to advancing GA, it is plausible that their FF remains relatively low for their specific gestational stage compared with uncompromised pregnancies. This persistent relative deficiency in FF likely reflects an underlying placental dysfunction that is not fully compensated for by the natural increase in FF over time. These pregnancies should not be equated with ‘normal’ FF pregnancies, as they may harbor placental dysfunction similar to persistently low FF cases and carry an elevated risk of pre‐eclampsia. Given the severity of pre‐eclampsia and its role in maternal/fetal mortality [15], clinicians must implement timely management strategies regardless of redraw success [16]. Strengthened antenatal monitoring and maternal assessment are crucial to prevent onset/progression and minimize risks.

The 3.5% FF threshold for NIPT failure used in this study is specific to the NIFTY platform [9]. As methodologies and bioinformatics vary across platforms, FF values and failure rates are not directly comparable, and this specific threshold may not be directly applicable to other assays that typically use thresholds of 2%–4% [3]. Notably, the optimal FF cutoff of 3.396% for pre‐eclampsia prediction, identified by our logistic regression model, aligns with both our technical threshold and the common industry range, suggesting a convergence between analytical and clinical thresholds. Future discrepancies in optimal cutoffs are likely to stem from methodological variations in FF quantification or from true biological differences in the patient populations studied. Clinically, an FF value at the lower end of a platform's reliable range—rather than a universal absolute cutoff—should be interpreted as a biomarker for placental pathology and an indicator of risk for pre‐eclampsia. Future multi‐platform studies to establish platform‐specific multiples of the median (MoM) for FF are warranted to standardize risk assessment.

Our findings are consistent with and further expand the existing body of evidence associating low FF with placental dysfunction. Previous studies, including that of Rolnik et al., have reported that low FF is associated with a higher risk of pre‐eclampsia [17], supporting the hypothesis that reduced FF may reflect placental size or early dysfunction. A key limitation of many previous studies was the lack of reliable outcome data. This gap has been addressed by large‐scale studies, such as the nationwide cohort study of 56,110 pregnancies by Becking et al., which found that low FF was associated with an increased risk of hypertensive disorders of pregnancy (adjusted odds ratio, 2.27) [18, 19]. Our study directly addresses the need for outcome data by providing follow‐up within the specific clinical context of initial NIPT failure, demonstrating lower FF in pregnancies that developed pre‐eclampsia. Accordingly, while our findings reinforce the established hypothesis, they provide critical complementary evidence by validating this association in the specific high‐risk context of initial NIPT failure.

The increasing use of XGBoost in clinical medicine demonstrates its potential to outperform conventional models [20, 21, 22, 23]. Our results confirm this and suggest that our XGBoost model is a promising adjunctive tool for early pre‐eclampsia diagnosis. To translate this into practice, careful integration into clinical workflows is required. First, a feasible implementation strategy would involve integrating the prediction algorithm into existing NIPT reporting software [24]. In this scenario, a NIPT failure due to low FF would automatically trigger the generation of a personalized risk score using readily available maternal traits (e.g., age, BMI, BP), appending this assessment directly to the clinical report [25]. This approach provides immediate, actionable insight without disrupting established workflows. Second, this tool should be positioned not as a replacement for first‐trimester screening but as a complementary, opportunistic screen. It uniquely leverages the common clinical occurrence of a failed NIPT to initiate risk assessment for a serious complication, thereby representing a novel strategy for secondary prevention. Finally, for pregnancies with an initial low FF, the model enables risk stratification to guide the intensity of prenatal surveillance. Pregnancies classified as high‐risk by the algorithm could be referred for more frequent blood pressure monitoring, placental growth factor (PlGF) testing, or earlier growth ultrasounds, while those with a low risk score might require less intensive follow‐up. Future prospective studies in larger cohorts are necessary to validate these risk thresholds and evaluate the clinical utility and cost‐effectiveness of this approach.

Additionally, two fetal chromosomal abnormalities occurred in Group A2, resulting in a significantly higher incidence compared with other groups. As male FF determination relies on Y‐chromosome reads, low FF in these cases may relate to fetal Y‐chromosome abnormalities [26, 27]. FF is influenced by chromosomal abnormalities: increased in trisomy 21, decreased in trisomy 18, trisomy 13, and monosomy X [28, 29]. The specific correlation between FF and Y‐chromosome abnormalities warrants further investigation.

This study has several strengths, including its prospective design and the application of a machine learning algorithm to a pressing clinical issue. Despite these strengths, important limitations must be considered. The limited number of pre‐eclampsia cases is a key constraint, common in studies of rare outcomes. Although this increases the risk of overfitting, rigorous internal validation confirmed stable model performance. Furthermore, the sample sizes for complications other than pre‐eclampsia are too limited in our single‐center cohort to support reliable models for each outcome. Finally, the absence of postpartum placental pathological examination means that our mechanistic inference is based on biological rationale from the literature.

Future large‐scale, multi‐center studies are essential to directly correlate antenatal FF levels with placental pathology, thereby providing definitive mechanistic evidence, and to develop integrated predictive tools for a broader spectrum of adverse pregnancy outcomes.

In conclusion, this study provides evidence that low FF is associated with an increased risk of pre‐eclampsia. Lower FF appears to be a valuable predictor of this complication. The XGBoost machine learning model predicted pre‐eclampsia with high accuracy, demonstrating its potential as a beneficial adjunct to prenatal screening for early diagnosis. NIPT failure due to low FF is an unavoidable aspect of current testing. While redraw is an option, success is not guaranteed. Therefore, healthcare providers must remain vigilant for the possibility of pre‐eclampsia in later pregnancy for all women with initial NIPT failure due to low FF, irrespective of redraw outcome.

## Funding

The work was supported by the National Natural Science Foundation of China (82372303).

## Ethics Statement

This study was approved by the Ethics Committee of the Second Affiliated Hospital and Yuying Children's Hospital of Wenzhou Medical University with the number of 2023‐K‐267–01.

## Consent

All participants provided written informed consent prior to the NIPT procedure. The consent form explicitly authorized the hospital to use its de‐identified test data for teaching and research purposes.

## Conflicts of Interest

The authors declare no conflicts of interest.

## Supporting information


Supporting Information S1

## Data Availability

The data that support the findings of this study are available on request from the corresponding author.
