# Supplementary material for: The Prediction of Pre‐Eclampsia Using Low Fetal Fraction in a Machine Learning Model
Source: Prenat Diagn. 2025 Nov 27;46(2):236–42. doi: 10.1002/pd.70033 (PMC12880958; doi:10.1002/pd.70033)
Supplement: Supplementary file 1 — Supporting Information S1 [file PD-46-236-s001.doc]

**Table S1. Hyperparameter search space for XGBoost model tuning.**

| Hyperparameter | Values Tested |
| --- | --- |
| max_depth | [3, 5] |
| n_estimators | [100, 200] |
| learning_rate | [0.1, 0.2] |
| min_child_weight | [1, 3] |

**Table S2. Performance metrics of the XGBoost model based on 1,000 bootstrap resamples.**

| Metric | Value | 95% Confidence Interval |
| --- | --- | --- |
| Mean Bootstrapped AUC | 0.956 | 0.868 to 1.000 |
| Accuracy | 0.944 | 0.861 to 1.000 |
| Sensitivity | 0.500 | 0.000 to 1.000 |
| Specificity | 0.971 | 0.906 to 1.000 |
| Brier Score | 0.037 | 0.003 to 0.082 |
